# Complete mitochondrial genome and phylogenetic analysis of *Sinocyclocheilus oxycephalus* (Cypriniformes: Cyprinidae)

**DOI:** 10.1080/23802359.2018.1438859

**Published:** 2018-02-15

**Authors:** Chunqing Li, Haitao Huang, Sifan Yang, Hao He, Qingyong Fu, Shanyuan Chen, Heng Xiao

**Affiliations:** a School of Life Sciences, Yunnan University, Kunming, China;; b National Demonstration Center for Experimental Life Sciences Education (Yunnan University) Yunnan University, Kunming, China

**Keywords:** Mitochondrial genome, *Sinocyclocheilus oxycephalus*, next-generation sequencing

## Abstract

*Sinocyclocheilus oxycephalus* is a freshwater cyprinid fish of high nutritional value, endemic to Shilin County, Southwestern China. In this study, we first sequenced the complete mitochondrial genome (mitogenome) of *S. oxycephalus*. The whole length of mitogenome is 16,585 bp, which contains 13 protein-coding genes, two ribosomal RNA genes, 22 transfer RNA genes, and a control region. The gene arrangement and structure is identical to other previously reported *Sinocyclocheilus* fishes. The overall base composition is 31.26% A, 16.42% G, 25.41% T and 26.90% C, with AT content of 56.67%. Phylogenetic analysis using mitogenomes of 13 cyprinid fishes showed that *S. oxycephalus* are closely related to *S. anophthalmus*, *S. grahami*, and *S. wumengshanensis*, and 11 *Sinocyclocheilus* species are grouped as a monophyletic clade with strong supports.

*Sinocyclocheilus oxycephalus* is a freshwater cyprinid fish of high nutritional value and distributes in the Bajiang River and Heilongtan Reservoir in Shilin County, Southwestern China, which belong to mainstream Nanpanjiang River system (Li et al. 1985, 1995; Zhao and Zhang 2009). Here, we first report the complete mitochondrial genome (mitogenome) of *S. oxycephalus*, which provides the basis for future study on its systematics and conservation.

In this study, *S. oxycephalus* samples were collected from Heilongtan Reservoir in Shilin county (24.77°N, 103.32°E), Yunnan province, Southwestern China. The voucher specimen was preserved in 95% ethanol and deposited in the Zoological Specimen Museum of Yunnan University under accession number YNUSO20160610002). Genomic DNA from muscle tissue was extracted by DNeasy Blood & Tissue Kit (QiaGen, Valencia, CA). The DNA library was constructed and sequenced with Illumina Miseq platform (Illumina, San Diego, CA). The complete mitogenome sequence of *S. oxycephalus* was assembled with A5-miseq v20150522 (Coil et al. 2015) and SPAdes (Bankevich et al. 2012). The complete mitogenome was annotated using the online program DOGMA (Wyman et al. 2004) and tRNAscan-SE (Lowe and Eddy 1997).

The complete mitogenome of *S. oxycephalus* was deposited in the GenBank database with accession number MG686610. The whole length of mitogenome is 16,585 bp, which contains 13 protein-coding (PCGs), two ribosomal RNA (rRNA), 22 transfer RNA (tRNA) genes, and a control region (D-loop), showing 94% identities to *S. grahami* mitogenome (GenBank: GQ148557) (Wu et al. 2010). The gene arrangement and structure were identical to other *Sinocyclocheilus* fishes (Wu et al. 2010; Chen et al. 2017; Li et al. 2017). The overall base composition is 31.26% A, 16.42% G, 25.41% T and 26.90% C, with AT content of 56.67%. The ND6 and eight tRNA genes (tRNA-Gln, tRNA-Ala, tRNA-Asn, tRNA-Cys, tRNA-Tyr, tRNA-Ser(UCN), tRNA-Glu, and tRNA-Pro) were encoded by the light strand, whereas the remaining genes were encoded on the heavy strand. All PCGs start with an ATG codon except for COI with start codon of GTG. Six PCGs (COI, ATP6, COIII, ND4L, ND5, and ND6) contain TAA as stop codons. Three PCGs (ND1, ND2 and ND3) end in TAG termination codon, while four PCGs (COII, ATP8, ND4 and CYTB) share incomplete stop codon T–. The 12S and 16S rRNAs are 953 bp and 1678 bp in length, respectively, located between tRNA-Phe and tRNA-Leu genes and separated by the tRNA-Val gene. Twenty-two tRNA genes can be folded into a typical cloverleaf structure and are in the range of 67–76 bp. The control region (933 bp) is located between tRNA-Pro and tRNA-Phe genes.

To present phylogenetic relationships among *S. oxycephalus* and other *Sinocyclocheilus* fishes, phylogenetic trees were reconstructed using MrBayes v3.2.5 (Ronquist and Huelsenbeck 2003) and RAxML v7.0.4 (Stamatakis 2006). The phylogenetic trees generated by two methods showed similar topology (Figure 1). The phylogenetic results revealed that *S. oxycephalus* was closely related to *S. anophthalmus*, *S. grahami*, and *S. wumengshanensis* in Yunnan Province, and that 11 *Sinocyclocheilus* species were grouped as a monophyletic clade with strong supports.

**Figure 1.**
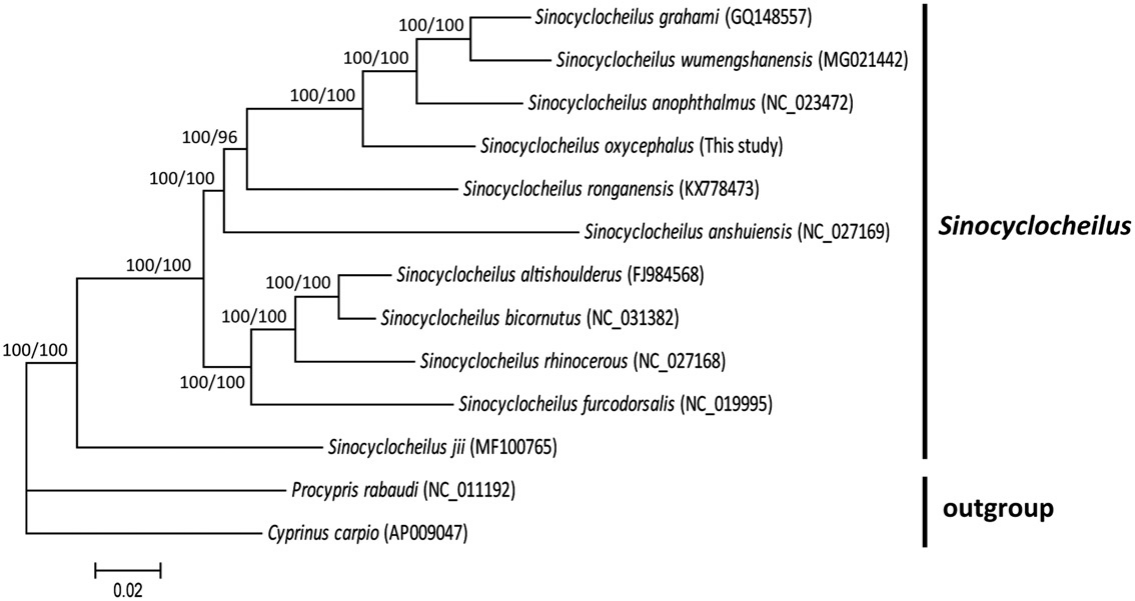
Phylogenetic tree of 11 *Sinocyclocheilus* fishes and two outgroups based on complete mitogenome sequences. The accession number for each species is indicated in bracket. The numbers above nodes represent posterior probability for Bayesian analysis and bootstrap value for ML analysis.

## References

[CIT0001] Bankevich A, Nurk S, Antipov D, Gurevich AA, Dvorkin M, Kulikov AS, Lesin VM, Nikolenko SI, Pham S, Prjibelski AD, et al 2012 SPAdes: a new genome assembly algorithm and its applications to single-cell sequencing. J Comput Biol. 19:455–477. 22506599 10.1089/cmb.2012.0021 PMC3342519

[CIT0002] Chen HY, Li CQ, Chen SY, Xiao H. 2017 The complete mitochondrial genome sequence and phylogenetic position of *Sinocyclocheilus wumengshanensis* (Cypriniformes: Cyprinidae). Mitochondrial DNA B: Resour. 2:821–822. 10.1080/23802359.2017.1407701 PMC7799495 33473996

[CIT0003] Coil D, Jospin G, Darling AE. 2015 A5-miseq: an updated pipeline to assemble microbial genomes from Illumina MiSeq data. Bioinformatics. 31:587–589. 25338718 10.1093/bioinformatics/btu661

[CIT0004] Li CQ, Xiang XH, Ning T, Chen SY, Xiao H. 2017 The complete mitochondrial genome sequence of *Sinocyclocheilus jii* (Cypriniformes: Cyprinidae) and phylogenetic implications. Mitochondrial DNA B: Resour. 2:638–639. 10.1080/23802359.2017.1375878 PMC7799893 33473929

[CIT0005] Li WX. 1985 Four new species of *Sinocyclocheilus* from Yunnan (Cypriniformes: Cyprinidae). Zool Res. 6:423–427.

[CIT0006] Li WX, Wu DF, Chen AL, Xu ZH. 1995 Analysis of nutrient composition of 2 *Sinocyclocheilus* in Yunnan. Chinese J Fisheries. 8:85–87.

[CIT0007] Lowe TM, Eddy SR. 1997 tRNA-scan-SE: a program for improved detection of transfer RNA genes in genomic sequence. Nucleic Acids Res. 25:955–964. 9023104 10.1093/nar/25.5.955 PMC146525

[CIT0008] Ronquist F, Huelsenbeck JP. 2003 MrBayes 3: Bayesian phylogenetic inference under mixed models. Bioinformatics. 19:1572–1574. 12912839 10.1093/bioinformatics/btg180

[CIT0009] Stamatakis A. 2006 RAxML-VI-HPC: maximum likelihood-based phylogenetic analyses with thousands of taxa and mixed models. Bioinformatics. 22:2688–2690. 16928733 10.1093/bioinformatics/btl446

[CIT0010] Wu XY, Wang L, Chen SY, Zan RG, Xiao H, Zhang YP. 2010 The complete mitochondrial genome of two species from *Sinocyclocheilus* (Cypriniformes: Cyprinidae) and a phylogenetic analysis within Cyprininae. Mol Bio Rep. 37:2163–2171. 19688279 10.1007/s11033-009-9689-x

[CIT0011] Wyman SK, Jansen RK, Boore JL. 2004 Automatic annotation of organellar genomes with DOGMA. Bioinformatics. 20:3252–3255. 15180927 10.1093/bioinformatics/bth352

[CIT0012] Zhao YH, Zhang CG. 2009 Endemic fishes of Sinocyclocheilus (Cypriniformes: Cyprinidae) in China: Species diversity, Cave adaptation, Systematics and Zoogeography. Beijing: Science Press.

